# Supplementary material for: Unexpectedly complex distribution pattern of chestnut pest Niphades castanea Chao (Coleoptera: Curculionidae) based on mtDNA and ITS markers
Source: PLoS One. 2024 Dec 12;19(12):e0310509. doi: 10.1371/journal.pone.0310509 (PMC11637356; doi:10.1371/journal.pone.0310509)
Supplement: S1 Table — (DOCX) [file pone.0310509.s001.docx]

**S1 Table Information on sample locations of chestnut pest *Niphades castanea*.**

| Pop names | Location | Latitude  (N) | Longitude  (E) | Altitude  (m) |
| --- | --- | --- | --- | --- |
| SL | Danfeng-Shangnan, Shaanxi province | 33.612°-33.778° | 110.57°-110.749° | 600-810 |
| BJ | Taibai-Zhenan-Meixian, Shaanxi province | 33.491°-34.13° | 107.507°-109.014° | 603.23-1296.5 |
| SMX | Sanmenxia, Henan province | 33.79° | 110.87° | 1121.9 |
| LY | Luanchuan, Henan province | 33.84° | 111.893° | 596.65-603.08 |
| XY | Fengdianxiang-Wuhexiang-Changzhuyuan-Tianpuxiang-Qianjinxiang, Henan province | 31.451°-31.795° | 114.72°-115.324° | 112.8-263.8 |
| JZ | Xiadianzi-Youfangdianxiang-Shibian-Liangtingao-Ligou-Yaotangcun-Changlingxiang, Anhui province | 31.231°-31.533° | 115.588°-115.994° | 158.92-613.3 |
| HS | Niujiaochong-Laohewan, Anhui province | 30.271°-31.339° | 116.346°-116.375° | 137.36-232.9 |
| YX | Banzhucun-Jiangjiawan-Youfangcun-Qianfota, Anhui province | 30.793°-30.952° | 116.073°-116.364° | 414.1-570.62 |
| XG | Baimaao, Hubei province | 31.65° | 114.541° | 100.1 |
| YS | Taohuachong-Guolutan, Hubei province | 30.938°-30.952° | 115.825°-116.22° | 158.27-493.36 |
| MC | Chengmagang-Anlecun, Hubei province | 31.347°-31.383° | 114.8°-114.938° | 132.7-154.3 |
| HA | Luojiawan, Hubei province | 31.484° | 114.779° | 117.3 |
| LT | Zaoshuao-Kuamadun-Jiuzihe, Hubei province | 30.782°-31.115° | 115.406°-115.684° | 147.82-293.26 |
| YC | Houpinglinchang, Hubei province | 31.203° | 111.063° | 1500.3 |
| DZ | Hongfeng, Sichuan province | 31.687° | 107.626° | 775.07 |
