# Supplementary material for: Unexpectedly complex distribution pattern of chestnut pest Niphades castanea Chao (Coleoptera: Curculionidae) based on mtDNA and ITS markers
Source: PLoS One. 2024 Dec 12;19(12):e0310509. doi: 10.1371/journal.pone.0310509 (PMC11637356; doi:10.1371/journal.pone.0310509)
Supplement: S2 Table — (DOCX) [file pone.0310509.s002.docx]

**S2 Table Fixation index (Fst) values among populations based on concatenated COI and COII gene sequences of *N. castanea*.**

|  | BJ | DZ | HA | HS | JZ | LT | LY | MC | SL | SMX | XG | XY | YC | YS |
| --- | --- | --- | --- | --- | --- | --- | --- | --- | --- | --- | --- | --- | --- | --- |
| BJ | 0.000 |  |  |  |  |  |  |  |  |  |  |  |  |  |
| DZ | -0.064 | 0.000 |  |  |  |  |  |  |  |  |  |  |  |  |
| HA | 0.420 | 0.959 | 0.000 |  |  |  |  |  |  |  |  |  |  |  |
| HS | 0.487 | 0.959 | 0.341 | 0.000 |  |  |  |  |  |  |  |  |  |  |
| JZ | 0.281 | 0.329 | -0.016 | 0.051 | 0.000 |  |  |  |  |  |  |  |  |  |
| LT | 0.441 | 0.891 | 0.122 | 0.404 | -0.018 | 0.000 |  |  |  |  |  |  |  |  |
| LY | -0.035 | 0.000 | 0.963 | 0.962 | 0.344 | 0.900 | 0.000 |  |  |  |  |  |  |  |
| MC | 0.551 | 0.974 | 0.695 | 0.778 | 0.018 | 0.255 | 0.976 | 0.000 |  |  |  |  |  |  |
| SL | 0.037 | -0.084 | 0.968 | 0.967 | 0.384 | 0.921 | -0.059 | 0.977 | 0.000 |  |  |  |  |  |
| SMX | -0.064 | 0.000 | 0.959 | 0.959 | 0.329 | 0.891 | 0.000 | 0.974 | -0.084 | 0.000 |  |  |  |  |
| XG | 0.071 | 0.229 | 0.034 | 0.132 | -0.034 | 0.080 | 0.273 | 0.227 | 0.397 | 0.229 | 0.000 |  |  |  |
| XY | 0.130 | 0.190 | 0.034 | 0.089 | 0.006 | 0.043 | 0.208 | 0.088 | 0.255 | 0.190 | -0.121 | 0.000 |  |  |
| YC | 0.763 | 1.000 | 0.992 | 0.991 | 0.646 | 0.832 | 1.000 | 0.989 | 0.999 | 1.000 | 0.632 | 0.480 | 0.000 |  |
| YS | 0.033 | 0.689 | 0.660 | 0.702 | 0.044 | 0.628 | 0.710 | 0.734 | 0.767 | 0.689 | -0.597 | -0.273 | 0.798 | 0.000 |
| YX | -0.136 | 0.675 | 0.473 | 0.513 | -0.360 | 0.414 | 0.685 | 0.542 | 0.716 | 0.675 | -8.514 | -0.633 | 0.879 | 0.097 |
