# Supplementary material for: Unexpectedly complex distribution pattern of chestnut pest Niphades castanea Chao (Coleoptera: Curculionidae) based on mtDNA and ITS markers
Source: PLoS One. 2024 Dec 12;19(12):e0310509. doi: 10.1371/journal.pone.0310509 (PMC11637356; doi:10.1371/journal.pone.0310509)
Supplement: S3 Table — (DOCX) [file pone.0310509.s003.docx]

**S3 Table Fixation index (Fst) values among populations based on ITS1 sequences of *N. castanea*.**

|  | BJ | DZ | HA | HS | JZ | LT | LY | MC | SL | SMX | XG | XY | YC | YS |
| --- | --- | --- | --- | --- | --- | --- | --- | --- | --- | --- | --- | --- | --- | --- |
| BJ | 0.000 |  |  |  |  |  |  |  |  |  |  |  |  |  |
| DZ | 0.029 | 0.000 |  |  |  |  |  |  |  |  |  |  |  |  |
| HA | 0.041 | 0.041 | 0.000 |  |  |  |  |  |  |  |  |  |  |  |
| HS | 0.012 | 0.183 | -0.013 | 0.000 |  |  |  |  |  |  |  |  |  |  |
| JZ | 0.118 | 0.486 | 0.186 | 0.023 | 0.000 |  |  |  |  |  |  |  |  |  |
| LT | 0.036 | 0.167 | -0.020 | -0.048 | 0.090 | 0.000 |  |  |  |  |  |  |  |  |
| LY | -0.076 | 0.169 | -0.077 | -0.067 | -0.094 | -0.051 | 0.000 |  |  |  |  |  |  |  |
| MC | 0.033 | 0.358 | 0.045 | 0.032 | -0.027 | 0.044 | -0.129 | 0.000 |  |  |  |  |  |  |
| SL | 0.055 | 0.381 | 0.076 | 0.033 | -0.004 | 0.058 | -0.073 | -0.005 | 0.000 |  |  |  |  |  |
| SMX | -0.004 | 0.180 | -0.066 | -0.051 | 0.055 | -0.068 | -0.044 | 0.056 | 0.037 | 0.000 |  |  |  |  |
| XG | -0.085 | 0.175 | -0.083 | -0.083 | -0.135 | -0.063 | 0.000 | -0.110 | -0.123 | -0.057 | 0.000 |  |  |  |
| XY | 0.169 | 0.614 | 0.224 | 0.159 | 0.003 | 0.187 | -0.008 | 0.028 | 0.034 | 0.201 | -0.139 | 0.000 |  |  |
| YC | 0.309 | -0.035 | 0.200 | 0.409 | 0.738 | 0.403 | 0.384 | 0.599 | 0.630 | 0.386 | 0.397 | 0.812 | 0.000 |  |
| YS | 0.048 | 0.233 | 0.038 | -0.047 | 0.011 | -0.032 | -0.108 | -0.028 | -0.014 | -0.080 | -0.121 | 0.025 | 0.512 | 0.000 |
| YX | 0.010 | 0.218 | -0.042 | -0.041 | 0.036 | -0.038 | -0.063 | 0.031 | 0.036 | -0.042 | -0.081 | 0.151 | 0.441 | -0.042 |
